# Oral Vaccination With Recombinant Vesicular Stomatitis Virus Expressing Sin Nombre Virus Glycoprotein Prevents Sin Nombre Virus Transmission in Deer Mice

**DOI:** 10.3389/fcimb.2020.00333

**Published:** 2020-07-08

**Authors:** Bryce M. Warner, Rohit K. Jangra, Bryan D. Griffin, Derek R. Stein, Darwyn Kobasa, Kartik Chandran, Gary P. Kobinger, David Safronetz

**Affiliations:** ^1^Department of Medical Microbiology and Infectious Diseases, University of Manitoba, Winnipeg, MB, Canada; ^2^Zoonotic Diseases and Special Pathogens, National Microbiology Laboratory, Public Health Agency of Canada, Winnipeg, MB, Canada; ^3^Department of Microbiology and Immunology, Albert Einstein College of Medicine, Bronx, NY, United States; ^4^Department of Pathology and Laboratory Medicine, University of Pennsylvania School of Medicine, Philadelphia, PA, United States; ^5^Department of Microbiology and Immunology, Faculty of Medicine, Laval University, Quebec City, QC, Canada

**Keywords:** sin nombre virus, hantavirus, hantavirus cardiopulmonary syndrome, deer mice, *Peromyscus maniculatus*

## Abstract

Sin Nombre virus (SNV) is the major cause of hantavirus cardiopulmonary syndrome (HCPS) in North America, a severe respiratory disease with a high fatality rate. SNV is carried by *Peromyscus maniculatus*, or deer mice, and human infection occurs following inhalation of aerosolized virus in mouse excreta or secreta, often in peri-domestic settings. Currently there are no FDA approved vaccines or therapeutics for SNV or any other hantaviruses, therefore prevention of infection is an important means of reducing the disease burden of HCPS. One approach for preventing HCPS cases is to prevent the spread of the virus amongst the rodent reservoir population through bait vaccination. However, bait style vaccines for rodent-borne viruses have not been employed in the field, unlike those targeting larger species. Here we utilized a recombinant vesicular stomatitis virus expressing SNV glycoprotein precursor (rVSVΔG/SNVGPC) in an attempt to prevent SNV transmission. Vaccination of deer mice with rVSVΔG/SNVGPC was able to reduce viral RNA copy numbers in the blood and lungs of directly infected animals. More importantly, vaccination, either intramuscularly or orally, significantly reduced the number of transmission events in a SNV transmission model compared with control animals. This provides a proof-of-concept in which oral vaccination of deer mice results in protection against acquiring the virus following direct contact with infected deer mice. Further development of bait style vaccines for SNV or other rodent-borne viruses could provide an effective means of reducing disease burden.

## Introduction

Hantaviruses are a family of enveloped, single stranded, negative sense RNA viruses that are part of the Order *Bunyavirales* (Jonsson et al., 2010). Hantaviruses have a global distribution, with two phenotypically different diseases caused in Eurasia and the Americas. In Europe and Asia, hantavirus infection causes hemorrhagic fever with renal syndrome, while in the Americas, they are the cause of hantavirus cardiopulmonary syndrome (HCPS) (Jonsson et al., 2010). HCPS can be caused by a number of different hantaviruses, but predominantly due to infection with Sin Nombre virus (SNV) in North America and by Andes virus (ANDV) and Araraquara virus (ARAV) in South America (Figueiredo et al., 2014; Drebot et al., 2015). HCPS is a severe respiratory disease with a case fatality rate as high as 35%. Disease is typified by general flu-like symptoms followed by sudden onset of cardiopulmonary involvement including cough, dyspnea, tachycardia, and then more severe symptoms such as pulmonary edema, bilateral infiltrates, hypotension, and cardiogenic shock resulting in mechanical ventilation and intensive care treatment. The incubation period averages 14–17 days and is followed by rapid deterioration of health and severe illness. Most hospital admission occur 3–6 days after the onset of symptoms, and the average time to death is within 2 days of hospital admission (Jonsson et al., 2010). Currently there are no FDA approved vaccines for prevention of hantavirus infection or therapeutics to treat HCPS.

Hantaviruses are zoonotic pathogens that can be carried by rodents, shrews, moles, or bats (Klempa et al., 2007; Jonsson et al., 2010; Kang et al., 2011; Weiss et al., 2012). Known pathogenic hantaviruses are carried by rodents, and the reservoir host for SNV is the deer mouse, *Peromyscus maniculatus* (Childs et al., 1994). Deer mice primarily become infected following direct contact with other infected deer mice, and infection persists throughout the lifetime of infected animals (Botten et al., 2003; Warner et al., 2019a). Human infection with SNV is caused by inhalation of aerosolized virus found in contaminated deer mouse excreta or secreta, usually in peri-domestic or field settings. Occupational hazards that increase the likelihood of exposure include farming, forestry, and cleaning of sheds, barns and cabins (Forbes et al., 2018). Cleaning of animal storage areas and sheds, seeding and plowing, handling and cutting firewood are all potentially high risk activities (Zeitz et al., 1995; van Loock et al., 1999; Vapalahti et al., 2010). Therefore, awareness and strong preventative measures in high risk situations are key to avoiding exposure.

One issue preventing the development and testing of vaccine candidates against New World hantaviruses is the relatively few cases of HCPS seen, particularly in North America. This makes vaccine efficacy studies difficult. Despite a number of various vaccine platforms that have undergone pre-clinical testing in animal models, and a vaccine in early clinical trials, a vaccine progressing through human trials remains unlikely (Brocato and Hooper, 2019). One approach for limiting the spread of zoonotic viral pathogens throughout their host populations is to employ vaccines targeting the wildlife population (Mendoza et al., 2018). This bait style vaccine approach has been successfully utilized against rabies virus in the US and Canada, effectively eliminating the virus among certain wildlife populations (Maki et al., 2017). Additionally, similar platforms have been developed and tested against other pathogens such as *Borrelia burgdorferi* and *Yersinia pestis* (Gomes-Solecki et al., 2006; Rocke et al., 2008). While bait style vaccines targeting smaller rodent populations have not been used extensively, this remains a potentially viable option for targeting specific populations within areas where there is a high risk of transmission to humans.

Here, we utilized a recombinant vesicular stomatitis virus expressing SNV glycoprotein precursor (rVSVΔG/SNVGPC), which has shown efficacy against SNV and ANDV in Syrian hamster models of infection (Warner et al., 2019b), to determine if vaccination of deer mice, either orally or intramuscularly, could prevent subsequent infection with SNV. Additionally, we wanted to determine whether this vaccination could prevent the acquisition of SNV in a SNV transmission model in deer mice (Warner et al., 2019a), mimicking a potential exposure situation following bait style vaccination. Our data show that vaccination was able to significantly reduce the risk of SNV infection following exposure, providing a proof-of-concept for the development of bait style vaccines for preventing the spread of rodent-borne viral pathogens such as hantaviruses.

## Results

**Figure 1:**
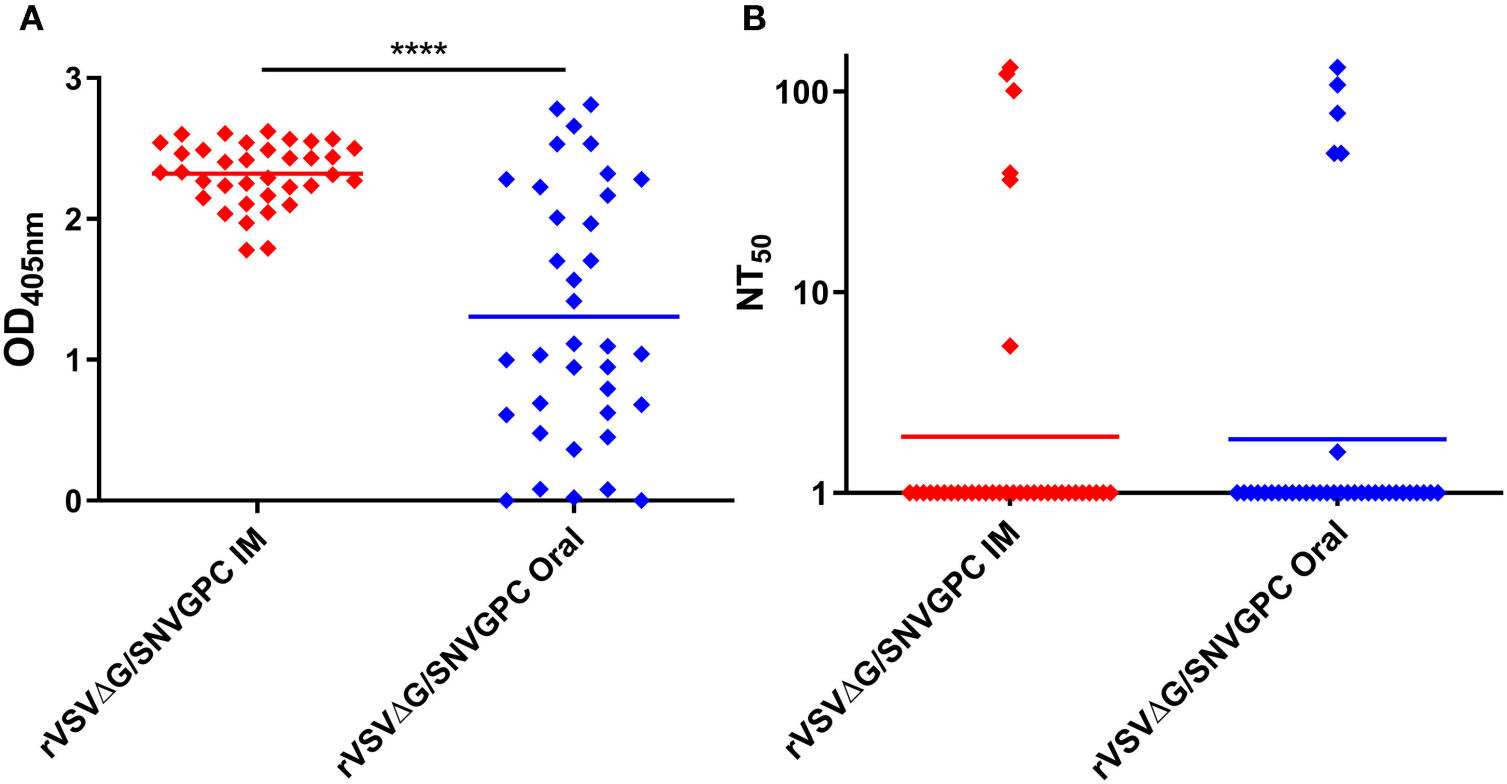
Humoral immune responses in deer mice vaccinated with rVSVΔG/SNVGPC. Deer mice were vaccinated with 2 × 10^4^ PFU of rVSVΔG/SNVGPC either IM or via oral gavage. After 28 days, sera were collected from vaccinated mice and the presence of **(A)** total anti-SNV IgG was detected, or **(B)** anti-SNV neutralizing antibodies were detected. Shown are data means + standard deviation *n* = 36. Data shown are combined from two separate experiments. Statistical significance assessed by Mann-Whitney test ****p < 0.0001.

**Figure 2:**
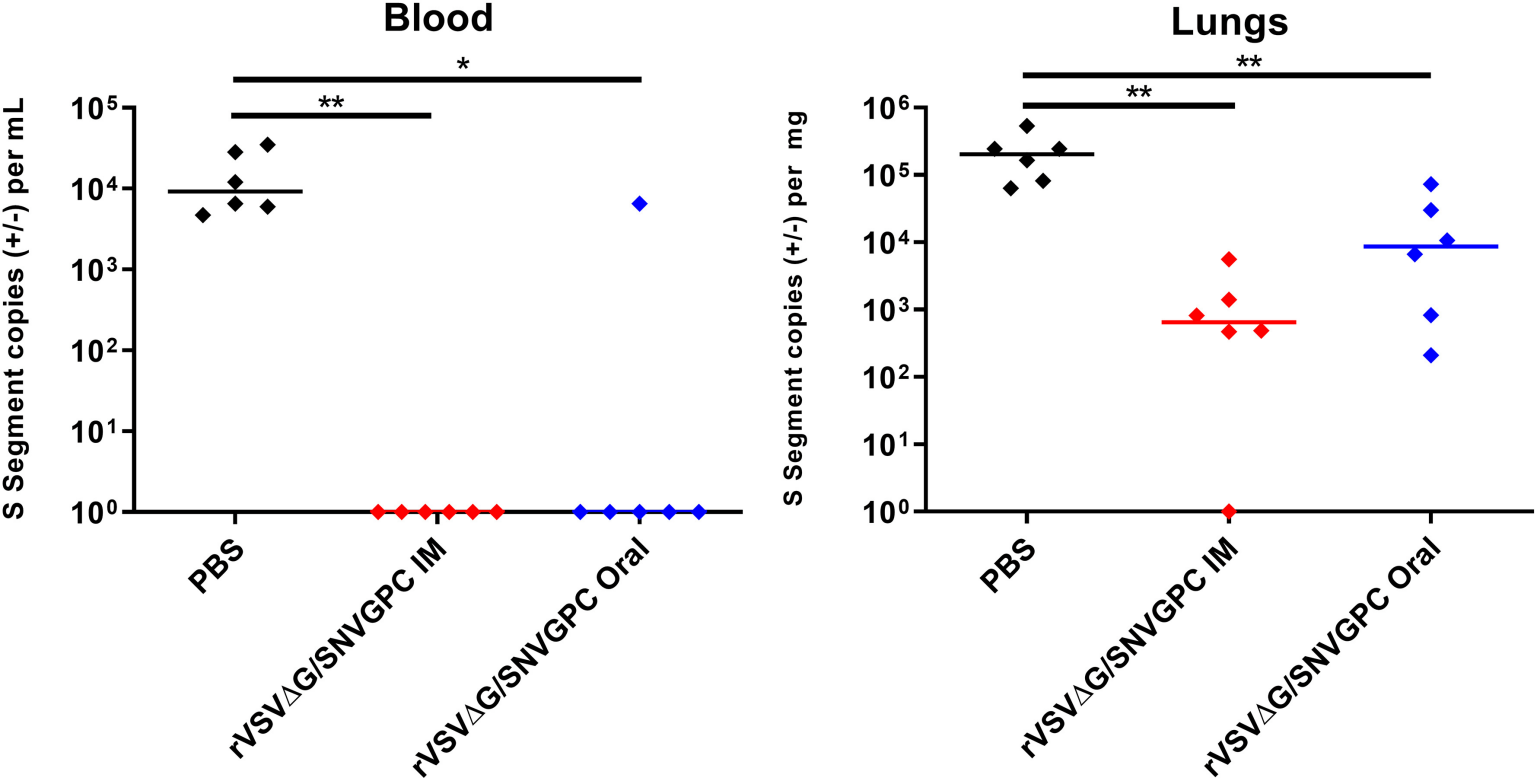
Protective efficacy of rVSVΔG/SNVGPC vaccination in deer mice. Vaccinated deer mice were infected with SNV 28 days following vaccination and SNV RNA levels were detected in the blood and lungs of infected mice 14 days post-infection. Shown are data medians. *n* = 6 Statistical significance assessed by Mann-Whitney test. *p < 0.05, **p < 0.01.

We wanted to determine whether vaccination with rVSVΔG/SNVGPC could protect deer mice against infection with SNV. We have previously shown that this vaccine is effective in hamsters and is able to protect against lethal ANDV infection as well as non-lethal hamster-adapted SNV (Warner et al., 2019b). Because the ultimate goal of vaccination of rodents is to prevent infection via bait style vaccines, we immunized deer mice with 2 x 10^4^ plaque forming units (PFU) of rVSVΔG/SNVGPC either intramuscularly or via oral gavage. rVSVΔG/SNVGPC immunization was significantly more immunogenic in terms of induction of SNV-specific IgG when administered intramuscularly as compared to oral delivery (Figure 1A). Neutralizing antibody titers in both groups of mice were very low, with only a small number of mice in each vaccinated group having detectable neutralizing antibody titers (Figure 1B). The low to non-existent neutralizing titers elicited by rVSVΔG/SNVGPC were surprising as this vaccine was able to induce a least low to modest neutralizing antibodies in hamsters (Warner et al., 2019b). This variability in antibody responses may have implications for vaccine efficacy. However, vaccination with a single dose of VSV-vectored vaccines does not always result in high neutralizing antibody titers, as seen with a VSV-vectored Lassa vaccine (Abreu-Mota et al., 2018). At 28 days after immunization, deer mice were then challenged intramuscularly with SNV to determine the protective efficacy of the vaccine. Challenged animals were euthanized 14 days later during what is the peak of acute infection and SNV RNA levels in the blood and lungs were quantified. Vaccination by either route was able to significantly reduce SNV RNA levels in both the blood and lungs (Figure 2). However, while significantly lower than in the PBS control group, rVSVΔG/SNVGPC vaccinated animals still had detectable SNV RNA in the lungs, suggesting that vaccination did not result in complete protective immunity. Although the animals vaccinated in this preliminary experiment were not completely protected against direct SNV infection, we went ahead and tested whether vaccination could prevent acquisition of SNV in a transmission model.

**Figure 3:**
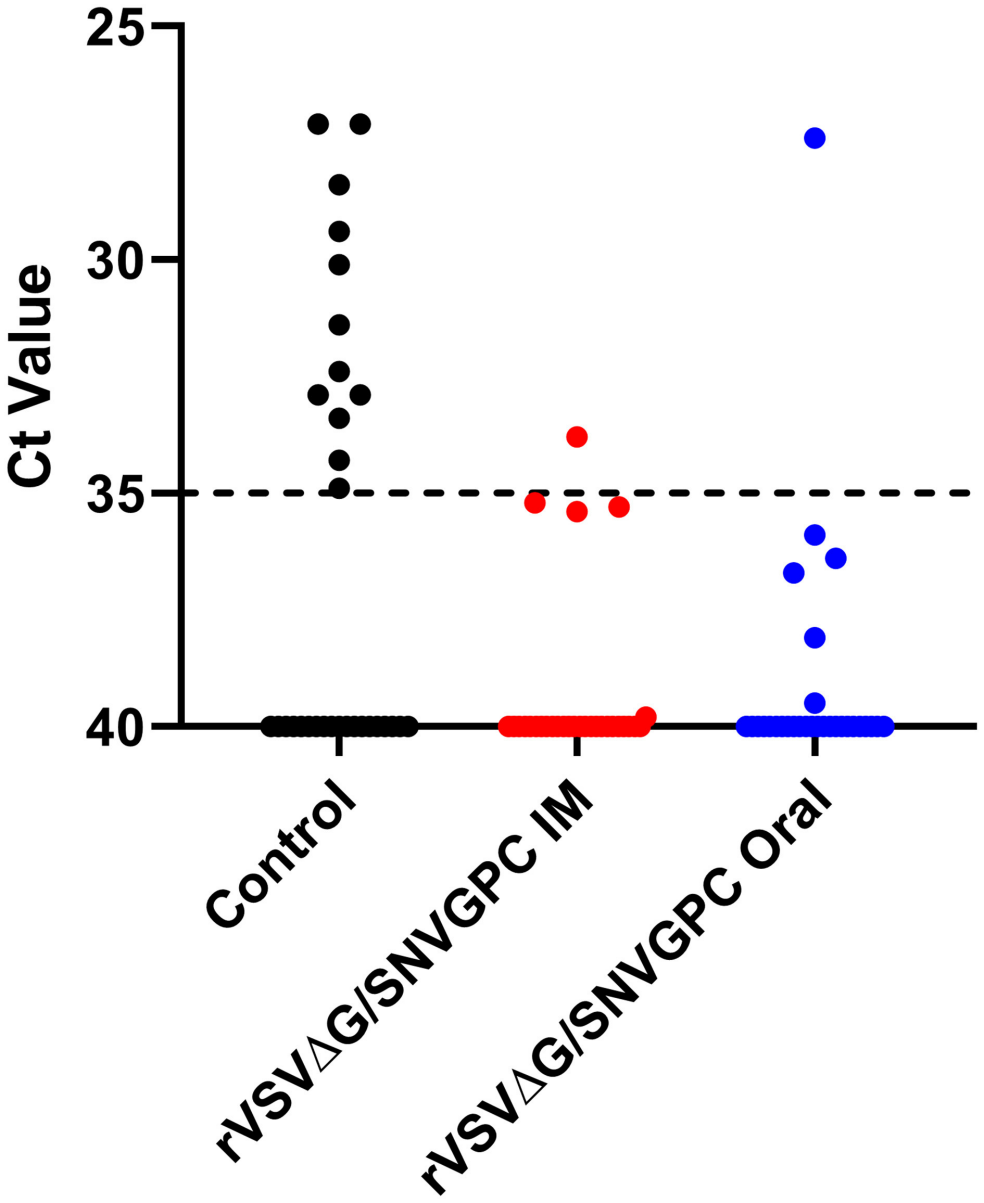
SNV RNA levels in the lungs of transmission experiment deer mice. Following 6 weeks of exposure to SNV infected deer mice, vaccinated, or control deer mice were euthanized and the presence of SNV RNA was detected in the lungs. Shown are Ct values from the lungs of individual deer mice *n* = 30 for IM and oral groups, 31 for controls.

Once again, deer mice were vaccinated with rVSVΔG/SNVGPC by either the intramuscular or oral route (*n* = 30). Twenty eight days following vaccination, serum samples were taken to examine the humoral immune responses induced in these mice. Intramuscular vaccination resulted in higher anti-SNV IgG titers compared with the oral group (Combined data in Figure 1A). Once again low levels of neutralizing antibodies were elicited by rVSVΔG/SNVGPC vaccination in both groups, with only a fraction of vaccinated animals showing detectable neutralizing antibodies (Figure 1B). On day 28 post-vaccination, rVSVΔG/SNVGPC vaccinated deer mice or unvaccinated control animals were moved into BSL-4 and housed with a deer mouse that was infected with SNV in a model of SNV transmission described previously by our group (Warner et al., 2019a). Following 6 weeks of direct exposure to the infected deer mice, all animals were euthanized to determine whether vaccination prevented transmission. In the unvaccinated control group, 12/31 exposed animals were either seropositive or had detectable SNV RNA in the lungs, while only 1/30 animals in each of the vaccinated groups became infected (Table 1, Figure 3). This represented a significant reduction in the risk of infection following exposure to infected animals [relative risk = 11.61 (1.607–83.92), *p* = 0.0011 (Fisher's exact test)] (Table 1). These data suggest that oral vaccination with rVSVΔG/SNVGPC or with other potential vaccine candidates can provide protection against acquiring SNV in deer mice.

**Table 1:** Prevention of transmission of SNV via vaccination.

**Group**	**Exposed, Naïve mice**	Transmission events*	**% of Naïve infected**	**Risk ratio (95% CI)**	***P* value (Fisher's exact test)**
Unvaccinated controls	31	12	39		
rVSVΔG/SNVGPC IM	30	1	3	11.61 (1.607–83.92)	0.0011
rVSVΔG/SNVGPC oral gavage	30	1	3	11.61 (1.607–83.92)	0.0011

## Discussion

Oral bait vaccines have been developed against several zoonotic pathogens and have the potential to eliminate or reduce the prevalence of a given pathogen within host populations. The most successful example of this approach is the use of vaccinia and Adenovirus based vectors for vaccinating various wildlife against Rabies virus (Rosatte et al., 2009; Fehlner-Gardiner et al., 2012; Maki et al., 2017). The use of similar vaccines for preventing the spread of rodent-borne diseases amongst wild rodent populations has not been studied as extensively. With a significant number of emerging and re-emerging viral pathogens such as hantaviruses and Lassa virus found in rodents which can infect humans, a bait vaccine approach could be an effective means of mitigating disease in high risk areas. We sought to determine whether the use of a vaccine targeting SNV could be utilized in this manner. We previously showed that the rVSVΔG/SNVGPC vaccine can provide protection in Syrian hamster models of ANDV and SNV infection (Warner et al., 2019b). This platform has been used extensively and has been approved for use in humans for vaccination against Ebola virus, highlighting its safety profile. We decided to test whether the same protection could be afforded to deer mice following vaccination, both by the classical intramuscular route or by oral delivery, which would be critical for bait vaccination. Despite low neutralization titers, rVSVΔG/SNVGPC vaccination was able to significantly reduce viral burden in the blood and lungs of infected deer mice (Figure 2). Due to the lack of a reliable assay for detecting live, replicating SNV it is difficult to determine whether the RNA detected represents viable, replicating SNV causing persistent infection of these deer mice. We went ahead and vaccinated groups of deer mice either intramuscularly or orally to determine whether this could prevent infection in an SNV transmission model.

Using our SNV transmission model, deer mice were vaccinated and then exposed to SNV infected deer mice by co-housing 28 days later. During 6 weeks of being co-housed with an infected deer mouse, only 1/30 vaccinated mice in each group became infected compared with 12/31 in the control group (Table 1). With our typical RT-qPCR threshold cutoff of 35, only a single animal in each of the intramuscular and oral vaccination groups was positive, however there were some animals that fell just below this threshold as seen in Figure 3, suggesting that some of these animals had low levels of SNV RNA at the time of euthanasia. The reduction in the number of infected animals represented a significant decrease in the risk of becoming infected following exposure to the infected animal. Surprisingly, oral delivery was as effective in preventing infection as intramuscular vaccination, suggesting that VSV based vaccines can provide protection when given through this route, which was been shown previously in protection studies using VSV-EBOV (Jones et al., 2007). Oral vaccination provided protection against acquisition of infection despite varying levels of anti-SNV IgG, suggesting other potential means of protection such as IgA or cell-mediated immunity induced by the vaccine. IgA may be important in the context of mucosal vaccination and immunity, however we were unable to assess IgA levels due to a lack of available reagents. It has been previously estimated that the basic reproduction number for SNV in deer mice is around 1.3, with a range of 0–4 (Kaplan et al., 2016). Therefore, for protective herd immunity within a deer mouse population the threshold required would be between 23 and 75% of the population being vaccinated. For the use of rVSVΔG/SNVGPC as a potential candidate for bait vaccine development, its immunogenicity and effectiveness when being delivered via this route, or through ingestion is of critical importance. Some issues to be addressed moving forward include the length of protection afforded by vaccination, particularly vaccine consumption by deer mice and also a protective dose range. The dose used here of 2 × 10^4^ PFU is relatively low, however in field experiments and deployment, strict dosing will not be able to be controlled, and thus an assurance of high enough doses of the vaccine is needed. The low dose used here likely reflects situations in the field in which mice will only ingest a portion of baits. The effectiveness at a low dose range is an important factor to consider in developing bait style vaccines. Overall, this data indicates that vaccination of deer mice, specifically oral vaccination can be an effective means of preventing acquisition of SNV following direct exposure to infected deer mice. Additionally, rVSVΔG/SNVGPC appears to be a viable candidate for further development and testing. Codon optimization for targeting of deer mice could be a means of increasing immunogenicity and thus efficacy and something that could be explored moving forward. This is also the first use of a laboratory-controlled transmission model for SNV or hantaviruses to test the efficacy of vaccines targeting reservoir hosts. We provide evidence that this is an appropriate model for testing various vaccine platforms and that this may be an effective method of testing vaccines against SNV. The use of this model provides evidence that similar systems could also be developed and used to test preventative measures against other rodent-borne viruses of importance for human health.

## Materials and Methods

### Animal Ethics Statement

All experiments described were carried out at the National Microbiology Laboratory (NML) of the Public Health Agency of Canada. Experiments were approved by the animal care committee at the Canadian Science Center for Human and Animal Health in accordance with the guidelines set by the Canadian Council on Animal Care. *Peromyscus maniculatus rufinus* (deer mice) used for all the experiments were provided by a breeding colony housed at the University of Manitoba. The University of Manitoba breeding colony was established with deer mice brought in from a previously established breeding colony at Rocky Mountain Laboratories in Montana, USA, which had been established from deer mice obtained from a breeding colony at the University of New Mexico (Botten et al., 2001). All the deer mice from the colony were seronegative and Sin Nombre virus-free.

### Viruses, Vaccinations, and Infections

rVSVΔG/SNVGPC and SNV strain 77734 have been described previously (Botten et al., 2000; Warner et al., 2019a). The SNV strain 77734 is the original genotypically matched SNV strain for the subspecies of deer mice used by our group. The virus was originally isolated from a single wild *P. maniculatus rufinus* and used for the inoculation of deer mice in the original description of the experimental SNV infection of this species (Botten et al., 2000). The virus has been passaged only *in vivo* within deer mice. Incoming animals were acclimated for at least 1 week before the experimental procedures began. Animal work and infections were performed under Biosafety level-2 (BSL-2) (vaccinations) and BSL-4 conditions (SNV infection and transmission) at the NML. The animals were given food and water *ad libitum* and monitored daily throughout the course of the experiments. For vaccinations, deer mice were given 2 x 10^4^ PFU of rVSVΔG/SNVGPC or PBS either intramuscularly (in 100 μL, 50 μL per leg in hind leg musculature) or via oral gavage (in 100 μL). At 28 days post-vaccination, deer mice were then either infected with the equivalent of 2 × 10^5^ genome copies of SNV for infection experiments or housed with unvaccinated animals that were given the same dose for transmission experiments.

### Sin Nombre Virus Transmission

The SNV transmission model used to assess vaccine efficacy has been described previously (Warner et al., 2019a). Briefly, vaccinated or unvaccinated control deer mice were housed with a single SNV-infected, sex-matched deer mouse for 6 weeks. One infected deer mouse was housed with three uninfected deer mice in all cages, except for a single cage in the control group which housed four uninfected mice. Six weeks post-infection, all the mice were euthanized, and blood and lung samples were taken to determine the presence of SNV and/or seroconversion.

### Detection of Viral RNA

Detection of SNV RNA in tissues was performed as described previously (Warner et al., 2019a). All mice were exsanguinated via cardiac puncture under isoflurane anesthesia before being euthanized. Either whole blood in K2-EDTA tubes or serum was collected. Samples of lung, heart, and spleen were collected in 1 mL of RNAlater for the detection of SNV RNA. After 24 h in RNAlater, the collected tissues were removed from RNAlater, homogenized in 600 μL RLT lysis buffer (Qiagen), clarified by centrifugation, and diluted to 30 mg equivalents in RLT lysis buffer. RNA was extracted using an RNeasy mini kit, per manufacturer's instructions (Qiagen). RNA from whole blood, was extracted using a Viral RNA mini kit as per manufacturer's instructions (Qiagen). RT-qPCR detection of SNV S segment was performed on a QuantStudio 3 instrument (Applied Biosystems, Foster City, CA, USA) using a one-step protocol using a Quantitect probe RT-PCR kit (Qiagen) per the manufacturer's instructions in triplicate (SNVforw—GCAGACGGGCAGCTGTG; SNVrev—AGATCAGCCAGTTCCCGCT; SNVProbe−5′FAM-TGCATTGGAGACCAAACTCGGAGAACTC-3′IAbkFQ). RT-PCR was carried out in 3 stages: reverse transcription (50°C for 30 min), Taq activation (95°C for 15 min), and amplification (40 cycles of 94°C for 15 s and 60°C for 60 s). Data acquisition occurred at the end of the annealing/extension stage (60°C for 60 s) of each amplification cycle. A standard curve ranging from 5 × 107 to five copies of *in vitro* transcribed SNV S segment RNA was used to calculate the copy number per mL or mg of tissue for each sample by interpolation. A Ct cut-off value of 35 was used for determining positive samples, as this Ct value corresponded to a copy number of <1.

### Determination of Seroconversion by Anti-nucleocapsid ELISA

Seroconversion was determined as described previously (Warner et al., 2019a). Ninety-six-well, half-area, high-binding polystyrene plates (Corning) were coated with recombinant SNV nucleocapsid protein at 30 ng per well and incubated overnight at 4°C. The following day, the plates were washed with PBS-T and then blocked with 5% skim milk in PBS-T (PBS + 0.1% Tween 20) for 1 h at 37°C. The serum samples were diluted 1:100 in 5% skim milk in PBS and added to PBS-T washed plates in triplicate overnight at 4°C. The following day the plates were washed with PBS-T and secondary HRP-conjugated anti-Peromyscus leucopus antibody (KPL; 1:1,000) was added to the plates for 1 h at 37°C. The plates were washed with PBS-T, and ABTS substrate (Thermofisher) was added and incubated for 30 min at room temperature before reading the OD values at 405 nm. Positive samples were those that had an OD greater than the mean OD plus 3 standard deviations seen in the negative control wells.

### Detection of Anti-SNV GPC Humoral Immune Responses

For detection of SNV glycoprotein-specific antibodies following immunization, 96-well half-area plates (Corning) were coated with purified, concentrated rVSVΔG/SNVGPC particles at 500 ng of protein per well overnight at 4°C. The following day, plates were washed three times with PBS-T and blocked for 1 h with 5% skim milk + 0.01% tween 20. Following blocking, plates were washed three times with PBS-T and deer mouse serum diluted 1:100 in blocking buffer was added to plates in triplicate and incubated at 4°C overnight. The next day, the plates were washed three times with PBS-T and secondary peroxidase-labeled anti-Peromyscus leucopus IgG was added to the plates (1:1,000) for 1 h at 37°C. Following three washes with PBS-T, 75 μL/well of one-step ABTS substrate (Thermofisher) was added to the plates for 30 min at room temperature. Plates were then read at 405 nm and analyzed using SoftMax Pro software (version 6.1). Reported are average OD_405nm_ values for each sample ran in triplicate minus the average OD_405nm_ values of the negative control/blank wells for that plate.

For determination of neutralizing antibody responses, dilutions of vaccinated deer mouse serum were incubated with recombinant VSV expressing SNV glycoprotein and green fluorescent protein (rVSVΔG/SNVGPC-GFP) for 1 h at 37°C and then the viruses were used to infect monolayers of VeroE6 cells in 96 well plates. The number of cells and wells expressing GFP were enumerated manually under fluorescence microscopy. Neutralizing titer 50, or NT50 was determined by the Reed-Meunch method as the titer of serum that was able to provide a 50% reduction in relative infection.

### Statistical Analysis

All the results were analyzed and graphed using Prism 5 software (Graphpad). The statistical significance between the groups was determined using a Mann–Whitney test or Fisher's exact test, where appropriate.

## Data Availability Statement

The datasets generated for this study are available on request to the corresponding author.

## Ethics Statement

The animal study was reviewed and approved by Animal Care Committee, Canadian Science Center for Human and Animal Health.

## Author Contributions

BW, BG, and DRS performed the experiments. BW wrote the manuscript. RJ and KC designed and developed the rVSVΔG/SNVGPC vaccine. GK, DK, and DS supervised all work, consulted on the scientific aspects of the work, and provided input on the content of the manuscript. All authors contributed to the article and approved the submitted version.

## Conflict of Interest

The authors declare that the research was conducted in the absence of any commercial or financial relationships that could be construed as a potential conflict of interest.
